# Myocardial Strain Imaging in Resistant Hypertension

**DOI:** 10.1007/s11906-021-01148-3

**Published:** 2021-05-05

**Authors:** Reem Alsharari, David Oxborough, Gregory Y. H. Lip, Alena Shantsila

**Affiliations:** 1 Institute of Cardiovascular Sciences, University of Birmingham, Birmingham, UK; 2 Liverpool Centre for Cardiovascular Science, University of Liverpool, Liverpool Heart & Chest Hospital and Liverpool John Moores University, Liverpool, UK; 3 College of Applied Medical Sciences, King Saud Bin Abdulaziz University for Health Sciences, Riyadh, Kingdom of Saudi Arabia; 4 Aalborg Thrombosis Research Unit, Department of Clinical Medicine, Aalborg University, Aalborg, Denmark

**Keywords:** Speckle tracking, Deformation imaging, Systolic dysfunction, Uncontrolled hypertension, Resistant hypertension, High blood pressure

## Abstract

**Purpose of Review:**

Resistant hypertension (RH) is a major contributor to cardiovascular diseases and is associated with increased all-cause and cardiovascular mortality. Cardiac changes such as impaired left ventricular (LV) function, left ventricular hypertrophy (LVH), myocardial fibrosis, and enlarged left atrium (LA) are consequences of chronic exposure to an elevated blood pressure. The purpose of this review article is to demonstrate the potential benefits of using STE as a non-invasive imaging technique in the assessment of cardiac remodeling in patients with hypertension and specifically in uncontrolled and RH population.

**Recent Findings:**

It is well-recognized that conventional transthoracic echocardiography is a useful analytic imaging modality to evaluate hypertension-mediated organ damage (HMOD) and in a resistant hypertensive population. More recently two-dimensional speckle tracking echocardiography (STE) has been utilized to provide further risk assessment to this population.

**Summary:**

Recent data has shown that STE is a new promising echocardiographic marker to evaluate early stage LV dysfunction and myocardial fibrosis over conventional 2D parameters in patients with cardiovascular diseases.

## Introduction

Hypertension (HTN) remains the leader of cardiovascular mortality among several risk factors [1]. It has been reported to be responsible for increased incidence of heart failure (HF), cardiovascular comorbidities, and stroke [2–5]. Despite advances in diagnosis and management strategies of HTN, uncontrolled HTN remains a challenging problem and is considered as a primary cause of death for 7.5 million people each year globally [6]. Resistant hypertension (RH) is defined as office systolic and diastolic blood pressure exceeding 140 mmHg and 90 mmHg, respectively, in spite of the concurrent use of three or more antihypertensive agents, one of which being a diuretic [7]. Patients with confirmed RH are estimated to experience 50% more cardiovascular events compared to controlled HTN [8].

Impaired left ventricular (LV) function, left ventricular hypertrophy (LVH), and myocardial fibrosis are recognized markers of target organ damage, compromised in patients with long standing HTN [9–11]. However, the relationship between HTN and cardiac remodeling is not completely identified [12]. Conventional two-dimensional (2D) echocardiography provides useful structural and hemodynamic findings that are potent predictors of poor prognosis associated with HTN. Speckle tracking echocardiography (STE) has emerged as a non-invasive and sensitive method for detection of early regional and global myocardial dysfunction that are undetected by conventional parameters in both symptomatic and asymptomatic patients with cardiovascular disease [13•]. This review aims to comprehensively assess the literature on potential benefits of STE use in the evaluation of cardiac remodeling in patients with uncontrolled HTN and RH.

## Principle of LV Function Quantification by Speckle Tracking Echocardiography

Myocardial strain refers to the percentage deformation of the myocardium during the cardiac cycle. It represents the extent of regional myocardial deformation in a specified period of time in three orthogonal directions (longitudinal, radial, and circumferential). All were determined by length, thickness, and shortening, using the formula ε = (L-Lο) / Lo, where ε indicates strain (has a unit of %), L indicates length after deformation, and Lo indicates baseline length. Strain rate (SR) refers to the speed at which the myocardium deforms (velocity changes/distance) [13•].

Initially, two techniques were introduced to assess myocardial strain: (i) cardiac magnetic resonance (CMR) in the late 1980s [14•] and (ii) tissue Doppler imaging (TDI) in the 1990s [15]. While TDI is considered a feasible and reliable technique, it has several limitations that still remain unresolved. TDI is highly angle dependent, is constrained to longitudinal cardiac deformation, and suffers from poor signal to noise ratio [16]. STE is a promising technique which was introduced in the early 2000s [17] and has been validated against sonomicrometry (which involves the implantation of piezoelectric crystals and measures of the changes in distance between embedded crystals, due to the myocardium movement) and tagged CMR [18, 19•]. It is used to assess myocardial function, and it overcomes the limitations of TDI [20].

**Fig. 1:**
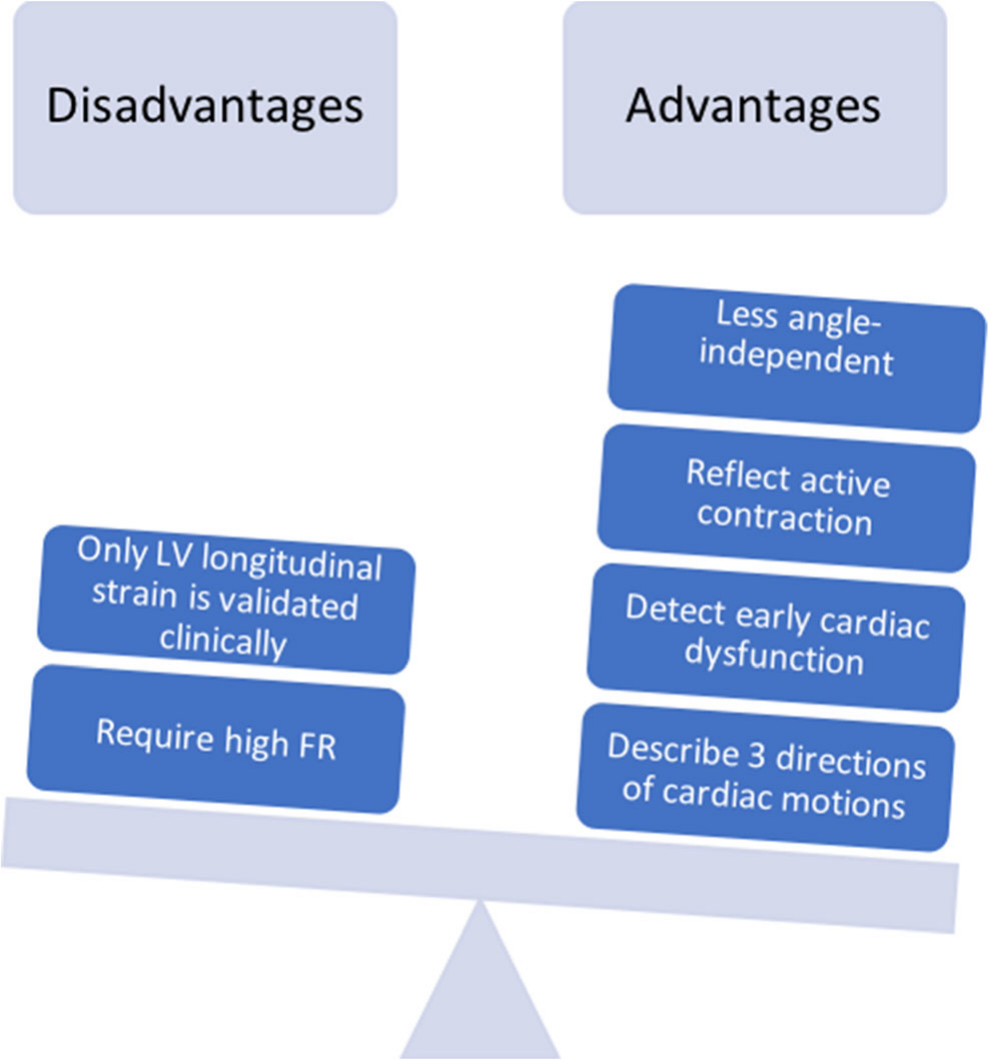
Speckle tracking echocardiography advantages. LV, left ventricular; FR, frame rate

The main advantage of STE is its ability to reflect active contraction within each segment, avoiding tethering effect, which makes it less influenced by artefacts. It can measure three directions of cardiac motion and can track the speckle in any 2D direction, making it less angle dependent (Fig. 1).

Heterogeneous ultrasound-myocardial tissue interactions produce an interference pattern, which is identified as a unique stable set of speckles [21]. STE modality identifies speckles based on echocardiographic images and tracks them between consecutive frames. It includes evaluation of myocardial strain, strain rate, and rotational deformation, which all are obtained by using specific software [22].

**Fig. 2:**
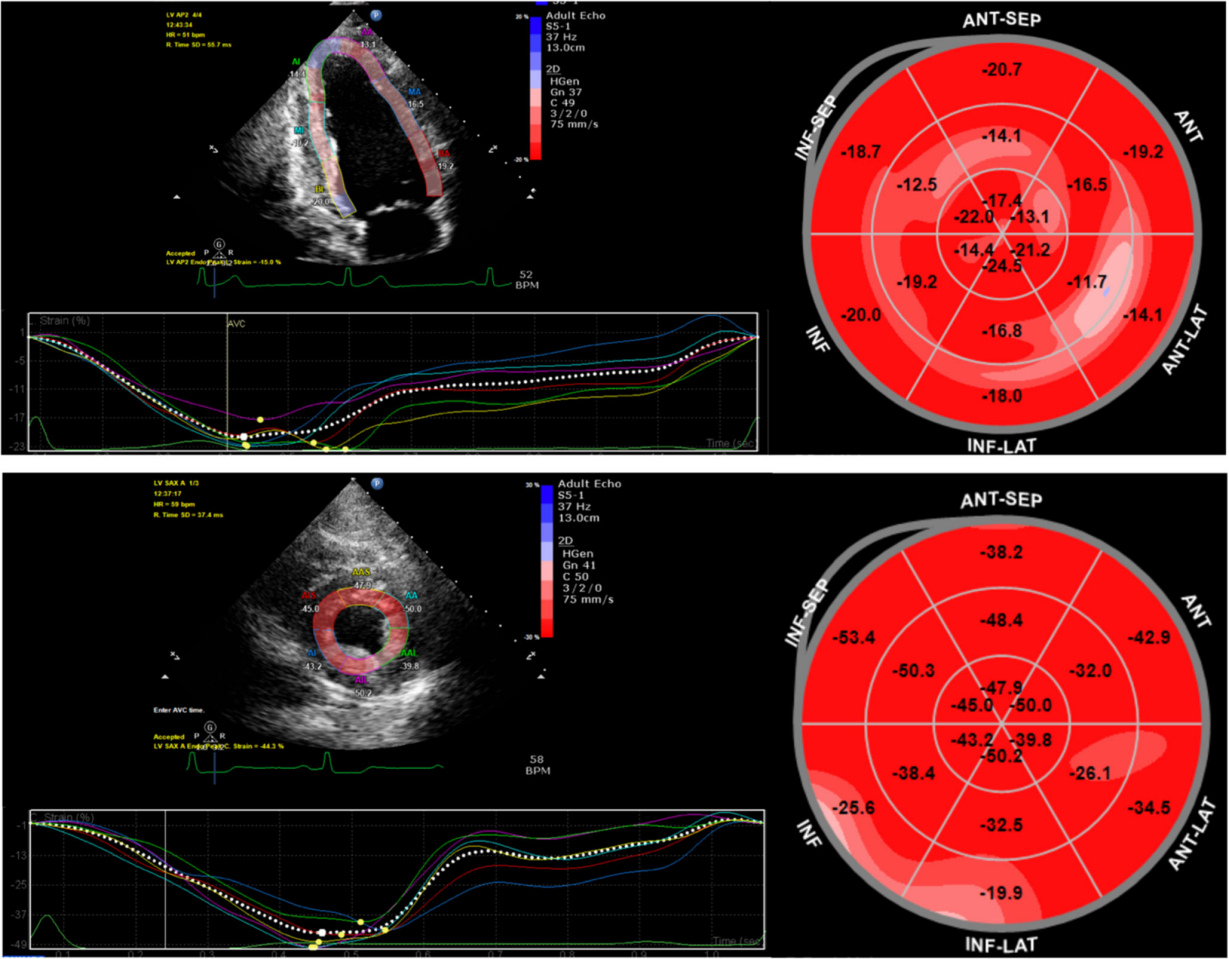
Example of GLS (upper) and GCS (lower) of LV. *GCS* global circumferential strain; *GLS* global longitudinal strain; *LV* left ventricular

Myocardial strain derived from STE can be measured in 3 planes. Circumferential and longitudinal strains represent a shortening of the LV cavity, and both have negative values (Fig. 2). Radial strain represents myocardial thickening of the LV in systole (secondary to the conservation of mass from longitudinal and circumferential shortening) and is denoted as a positive value. All strain parameters can be evaluated globally or regionally. Global longitudinal strain (GLS), global circumferential strain (GCS), and global radial strain (GRS) are calculated as an average of segmental regional strain. The average normal GLS is − 19.7% [23•], with a borderline level of − 18% [23•, 24]. Normal GCS is considered to be between − 20.9 and − 27.8%, and average GRS is between 35.1 and 59.0% [23•]. STE also provides the capacity to measure twist and torsion which are the parameters to determine deformation of LV [17].

## The Role of Strain in Predicting Early Damage in Hypertension

Conventional echocardiography is a reliable method widely used to detect impaired LV systolic and diastolic function in HTN. It is also used to calculate LV mass and determine the presence and the degree of LVH, a predictor of morbidity and mortality in HTN [25, 26].

However, it has been shown that HTN is associated with reduction in LV systolic strain in asymptomatic patients with normal ejection fraction (EF) with and without LVH, suggesting that LV mechanical abnormalities precede the development of LVH [27–29].

### Decreased Longitudinal Function in Hypertension

Normal myocardium consists of cardiac myocytes (30–40%) and non-myocyte components (60–70%) [30]. Myocardial fibers in the subendocardial layer are oriented in a longitudinal direction which then gradually change to a transverse direction in the middle layer and revert to longitudinal in the subepicardial layer [30].

Recent studies [28, 31–33] have closely linked the presence of fibrosis to attenuated myocardial strain. Cardiac remodeling in HTN involves an imbalance in the production of collagen types I and III (these subtypes are the major stress-bearing element within the ECM). This leads to an excessive deposition of collagen fibers in fibroblasts which transdifferentiate into myofibroblasts leading to heterogeneous acceleration of myocardial fibrosis [34, 35]. Moreover, it has been reported that increased matrix metalloproteinase-1 (MMP-1) turnover lead to reduced collagen I and III degradation and development of subendocardial myocardial fibrosis. This implies that irregular collagen production and myocardial fibrosis are associated with reduced GLS in HTN and hypertrophic cardiomyopathy [28, 31] and eventually lead to early impairment of systolic function [28, 32, 33]. Another pathway leading to activation of subendocardial production of collagen in HTN is pressure overload and high-end-systolic wall stress. The process involves collagen network thickening [36] and fibrosis build up primarily in the subendocardial layer.

Furthermore, fibrosis may have a possible direct effect on the rearrangement of myocardial sheets in subendocardial layers [30, 37] where maximum shearing deformation occurs, compared to the other layers [38, 39]. There is limited information available linking cardiac shear motion and systolic function.

### The Additive Value of Global Longitudinal Strain

Longitudinal, circumferential, and radial dysfunction do not occur in tandem with longitudinal subendocardial fibers being prone to being compromised first in several pathologies [40, 41]. GLS is the most widely used clinical application of STE. It has been recommended by the American Society of Echocardiography (ASE) for evaluation of global LV systolic function [13•] and has been widely validated [42, 43]. It is considered a strong indicator of an early phase of myocardial impairment in HTN as shown in Table 1 [29, 44–51]. It has been shown in some studies that the prevalence percentage of impaired GLS in hypertensive population vary between 15 and 42% [44, 52–54], suggesting for the influence of other related factors such as age, gender [55, 56], ethnicity, duration of the HTN, uncontrolled HTN [52–54], diabetes, and obesity [44].

**Table 1:** Summary of studies using a two-dimensional speckle tracking analysis in hypertensive populations

Author/year	Methods	Patient population	Sample size	STE software/echo machine	STE parameters	Follow-up period	Results
Bendiab et al., 2017 [44]	2D STE	HTN/overweight HTN/diabetes HTN/dyslipidemia Uncontrolled HTN	200	EchoPAC, GE	GLS	1 year	↓GLS in uncontrolled HTN ↓GLS in long lasting HTN (> 10 years)
Saito et al., 2016 [45]	2D STE	HTN without ischemic heart disease	388	TomTec, GE	GLS	4 years	↓GLS predicts MACE
Lee et al., 2016 [46]	2D STE	HTN	95	EchoPAC, GE	Subendocardial LS Subepicardial LS	7.3 ± 2.0 years	↓ subepicardial LS Preserved subendocardial LS
Chen et al., 2016 [47]	2D STE	Controlled HTN (group 1) Uncontrolled HTN (group2) Healthy control (group 3)	361	QLAB, Philips	cEss MWFs LS CS RS	3 months	↓ myocardial function in group 2 vs. groups 1 and 3
Cheng et al., 2014 [48]	2D STE	Intensive treatment with SBP target < 130 mmHg (group 1) Standard treatment with SBP target < 140 mmHg (group 2)	182	TomTec	GLS	24 weeks	After therapy: ↑ GLS in group 1 ↑ GLS in lower BMI ↑ GLS in women
Dobrowolski et al., 2014 [49]	2D STE	RH OSA^–^/MS^–^ (group 1) OSA^+^/MS^–^ (group 2) OSA^–^/ MS^+^ (group 3) OSA^+^/MS^+^ (group 4)	155	EchoPAC, GE	GLS	-	↓ GLS in group 4 vs. groups 1, 2, and 3
Imbalzano et al., 2011 [10]	2D STE	HTN/LVH (group 1) HTN/no LVH (group 2) Healthy control (group 3)	102	EchoPAC, GE	GLS GCS GRS	-	↓ GLS in groups 1 and 2 vs. group 3

*2D STE* Two-dimensional speckle tracking echocardiography, *AFI* automatic function imaging, *BMI* body mass index, *cESS*, circumferential end-systolic wall stress, *CS* circumferential strain, *EF* ejection fraction, *GCS* global circumferential strain, *GE* general electric, *GLS* global longitudinal strain, *IVSDd* interventricular septal diastolic diameter, *LS* longitudinal strain, *LVH* left ventricle hypertrophy, *MACE* major adverse cardiac events, *MWFS* mid-wall fraction shortening, *MS*^*–*^ without metabolic syndrome, *MS*^*+*^ with metabolic syndrome, *OSA*^*–*^ without obstructive sleep apnea, *OSA*^*+*^ with obstructive sleep apnea, *PWDd* posterior wall diastolic diameter, *RDN* renal denervation, *RH* resistant hypertension, *RS* radial strain, *RWT* relative wall thickness, *↓* significant reduction, *↑* significant increase

Studies have shown that GLS might be beneficial as an independent predictor of cardiovascular outcomes in general population [57–59] and in a population with a wide range of EF [60]. It is a strong predictor of major adverse cardiac events (MACE) including HF, stroke, myocardial infraction (MI), and all-cause mortality [45, 61, 62]. In the Copenhagen City Heart Study [62], which includes 1296 of participants from general population, who underwent STE assessment between 2001 and 2003 and were followed until 2013, GLS was an independent predictor of cardiovascular death and morbidity, including HF and MI with a hazard ratio of 1.12 [1.08–1.17] (*p* < 0.001 per 1% decrease). This association persisted after multivariable adjustment for the following parameters: (age, gender, heart rate, HTN, systolic blood pressure, LVEF, LV mass index, LV dimension, deceleration time, LA dimension and E/e). [62] Similarly, Saito et al. [45] retrospectively collected data on MACE (all-cause death and admission because of HF, MI, and strokes, with a median follow-up 4 years) in asymptomatic non-ischemic subjects with high blood pressure. It has been shown that MACE occurrence was independently associated with greater incidence of concentric hypertrophy and reduced GLS (both *p* < 0.01).

Cheng et al. [48] examined whether systolic dysfunction assessed by STE improved by intensive antihypertensive treatment in 182 patients with uncontrolled HTN. The study assessed GLS before and after 24 weeks of antihypertensive treatment and showed an improvement in GLS in response to the treatment that was independent of changes in blood pressure and associated with increased dose. This is more likely to occur when afterload reducing treatment is used, which improves LV function independent of blood pressure readings [63]. Moreover, GLS improved by -1.4% more in uncontrolled hypertensive not meeting RH criteria females compared to uncontrolled hypertensive males (*p* = 0.003). This difference in the responses between the two genders could be due to the differences in GLS baseline values, where females had higher GLS compared to males. In addition, the association between female gender and improvement in GLS is unclear and has yet to be examined in the general population to confirm sex differences associated with LV function. Another observation found an improvement in GLS by -0.46% for every 5 kg/m^2^ reduction in body mass index (BMI) (*p* = 0.015).

Similar findings have been reported by other studies [64, 65] which links attenuated GLS with metabolic disorders and obesity. RESOLVE trail [64] examined participants with metabolic syndrome and showed reduced GLS compared to a control group, while Wong et al. [65] have shown that insulin and BMI are significantly and independently associated with strain function in obese population. However, in Crendal et al.’s study [64], 78% of participants had HTN which may consider as a confounding factor and could mask the actual association. In addition, 17% of participants were treated with beta-blockers, which have an established effect on LV remodeling.

### Circumferential and Radial Function

Notably, the mid-myocardial layer may remain unchanged or even increased compared to the longitudinal function, which probably explain the well-preserved function reflected by EF [29, 50, 51, 66] [67, 68]. Preserved radial and circumferential function at early stages of HTN linked to the cross-fiber shortening phenomenon from hypertension-related ventricular remodeling, where mid-wall myocardial fibers are not compromised and consequently circumferential and radial function are preserved [54]. Although this explanation has received reasonable attention, other theories suggest that reduced longitudinal and circumferential strain exists with preserved EF secondary to increased LV wall thickness [68].

However, longitudinal function is not always the earliest predictor in all circumstances. Previous studies have reported that all three planes of function (longitudinal, radial, and circumferential) may decline in HF, signifying a decompensation mechanism of the LV and impaired myocardial layers as a response to increase myocardial wall stress and disease progress [66, 69, 70]. This happens because the impairment of longitudinal function occurs in the earlier phases of the remodeling, followed by decrease in radial and circumferential function, which is associated with further LV dilatation leading to HF [71–73].

### Twist and Torsion Deformation

Rotation, twist, and torsion are several terms to describe additional deformation of the LV caused by the helical arrangement of myocardial fibers. LV rotation is defined as an apical counterclockwise movement and basal clockwise movement in systole. During systole, the LV stores potential energy, which is subsequently released in early diastole. Twist and untwist play an important role by storing and releasing this energy which leads to LV diastolic relaxation and early diastolic filling. Twist/untwist (°) and rate (°/s) are calculated as the net difference between basal clockwise and apical anticlockwise rotation and rotation rate [17]. Torsion is calculated by dividing the twist angle by apical-basal distance and measured in °/cm [17]. In a non-diseased population, LV twist is approximately 15° with apical rotation being between 5° and 10° (counterclockwise) and basal rotation between -4° and -7° (clockwise) as observed in studies by CMR tagging [74]. A study by Dong et al. [75] showed that as with other indices of cardiac function, rotation is affected by loading condition (preload and afterload) of LV. Rotation increases with increased preload (end-diastolic volumes) and decreased afterload (end-systolic volumes) [75]. Reduced LV untwisting, elevated torsion, and twist have been observed in hypertensive patients [10, 76–78] and in various cardiovascular diseases [79, 80]. Alterations of myocardial twist are also linked to aging. Previous studies have demonstrated decreased diastolic untwisting, increased LV rotation, and twist with age in a normal population [81].

## Conclusion

Myocardial fiber orientation is a fundamental feature of the myocardium, and it has a substantial role in systolic function. STE imaging is a new non-invasive cardiovascular imaging modality that can be used in clinical practice to understand the mechanism of cardiac deformation, particularly in patients with early compensation of myocardial function and in patients with RH. Using STE also offers comprehensive evaluation to detect the underlying impaired systolic function in several pathologies, including HTN, to deliver optimal management plan. Furthermore, this powerful and valuable technique provides accurate and objective measures on global/regional contractile function.

## Data Availability

Not applicable
